# Three-Dimensional Culture of Ameloblast-Originated HAT-7 Cells for Functional Modeling of Defective Tooth Enamel Formation

**DOI:** 10.3389/fphar.2021.682654

**Published:** 2021-06-02

**Authors:** Anna Földes, Thanyaporn Sang-Ngoen, Kristóf Kádár, Róbert Rácz, Ákos Zsembery, Pamela DenBesten, Martin C. Steward, Gábor Varga

**Affiliations:** ^1^ Department of Oral Biology, Semmelweis University, Budapest, Hungary; ^2^ Department of Orofacial Sciences, University of California San Francisco, San Francisco, CA, United States; ^3^ School of Medical Sciences, University of Manchester, Manchester, United Kingdom

**Keywords:** HAT-7, ameloblast, amelogenesis, bicarbonate transport, intracellular pH, calcium signaling, tight junction, fluoride

## Abstract

**Background:** Amelogenesis, the formation of dental enamel, is well understood at the histomorphological level but the underlying molecular mechanisms are poorly characterized. Ameloblasts secrete enamel matrix proteins and Ca^2+^, and also regulate extracellular pH as the formation of hydroxyapatite crystals generates large quantities of protons. Genetic or environmental impairment of transport and regulatory processes (e.g. dental fluorosis) leads to the development of enamel defects such as hypomineralization.

**Aims:** Our aims were to optimize the culture conditions for the three-dimensional growth of ameloblast-derived HAT-7 cells and to test the effects of fluoride exposure on HAT-7 spheroid formation.

**Methods:** To generate 3D HAT-7 structures, cells were dispersed and plated within a Matrigel extracellular matrix scaffold and incubated in three different culture media. Spheroid formation was then monitored over a two-week period. Ion transporter and tight-junction protein expression was investigated by RT-qPCR. Intracellular Ca^2+^ and pH changes were measured by microfluorometry using the fluorescent dyes fura-2 and BCECF.

**Results:** A combination of Hepato-STIM epithelial cell differentiation medium and Matrigel induced the expansion and formation of 3D HAT-7 spheroids. The cells retained their epithelial cell morphology and continued to express both ameloblast-specific and ion transport-specific marker genes. Furthermore, like two-dimensional HAT-7 monolayers, the HAT-7 spheroids were able to regulate their intracellular pH and to show intracellular calcium responses to extracellular stimulation. Finally, we demonstrated that HAT-7 spheroids may serve as a disease model for studying the effects of fluoride exposure during amelogenesis.

**Conclusion:** In conclusion, HAT-7 cells cultivated within a Matrigel extracellular matrix form three-dimensional, multi-cellular, spheroidal structures that retain their functional capacity for pH regulation and intracellular Ca^2+^ signaling. This new 3D model will allow us to gain a better understanding of the molecular mechanisms involved in amelogenesis, not only in health but also in disorders of enamel formation, such as those resulting from fluoride exposure.

## Introduction

Amelogenesis, the formation of dental enamel, is well understood at the histomorphological level, but the underlying molecular mechanisms are poorly characterized (Lacruz et al., 2017; Varga et al., 2018). Ameloblasts, derived from the oral epithelium, are known to secrete proteins and Ca^2+^ into the enamel extracellular matrix. They also regulate extracellular pH as the formation of hydroxyapatite crystals generates large quantities of protons that must be neutralized by HCO_3_^−^ secretion to allow continued crystal growth. Genetic or environmental impairment of transport and regulatory processes (e.g. dental fluorosis) leads to the development of enamel defects such as hypomineralized enamel (Lacruz et al., 2017; Varga et al., 2018).

Ameloblast differentiation and ion transport activities have primarily been investigated by immunohistochemistry and characterization of the enamel phenotypes of knockout mouse models. However, to understand the mechanisms by which environmental factors, such as exposure to high levels of fluoride, affect enamel formation, *in vitro* model systems are needed (Klein et al., 2017).

Culture of primary enamel organ epithelial cells results in two distinct cell shapes: either polygonal or stellate (DenBesten et al., 2005). Stellate cells, which have a phenotype similar to mouse LS8 cells, grow rapidly in 2D culture but cannot be maintained in 3D Matrigel (Li et al., 2006). Polygonal cells are similar in morphology to both ameloblast-like mouse ALC (Nakata et al., 2003) and rat HAT-7 (Kawano et al., 2002) cells. The primary, enamel-originated cells grown in Matrigel alter their morphology in the presence of extracellular calcium (Li et al., 2006; Yan et al., 2006; Chen et al., 2009; He et al., 2010). However, these primary ameloblast-like cells can be passaged only 3 times, and so have limited use in studies of ameloblast function. When transfected by SV40 or telomerase (He et al., 2010; Zheng et al., 2013b; Zheng et al., 2013c), they maintain ameloblast-like characteristics through approximately eight passages (unpublished results), but do not form spheroid structures in Matrigel (Li et al., 2006). This then suggests that these transfected cells are also not a robust model to study ameloblast function (He et al., 2010; Zheng et al., 2013b; Zheng et al., 2013c). While these early studies offer the promise of advanced technologies to more completely regenerate the enamel organ epithelium, further advances are needed to identify cells that retain characteristics similar to primary cells, and that can be scaled up, expanded and standardized. This in turn will allow studies of tooth formation *in vitro*, and investigation of the mechanisms responsible for environmental effects on tooth enamel formation (Klein et al., 2017).

Scaling up of stem cells and progenitors can be performed using three different strategies: 1) monolayer cultures, 2) culturing cells on microspheres and 3) organoid/spheroid production (Silva Couto et al., 2020). Monolayer cultivation is the least effective method. To achieve higher yields of cell expansion, we can use microcarriers, which are principally designed for growing adherent cells in suspension culture systems. The application of microcarriers in dynamic culture systems may provide high yields but it is technically difficult to isolate the cells from their microcarriers (Chen et al., 2015; Lam et al., 2017; Cherian et al., 2020). In organoid/spheroid culture, three-dimensional cell aggregates can be produced under either static or dynamic conditions. This approach is thought to more closely mimic the three-dimensional *in vivo* environment (Wang et al., 2009; Bartosh et al., 2010). An obvious challenge of this strategy is to expand cells under controlled conditions without causing harmful effects on the cells and undesirable cell differentiation (Simoes et al., 2013; Yang et al., 2018a).

In the present study, we have investigated the effects of culture conditions on the three-dimensional growth of HAT-7 cells. These cells, which are derived from rat incisor cervical loop (Kawano et al., 2002), maintain a polygonal shape and exhibit characteristics of maturation-stage ameloblasts, such as the expression of the marker proteins kallikrein-4 and amelotin (Kawano et al., 2002). We have shown previously that HAT-7 cells grown on permeable supports form two-dimensional polarized epithelia which serve as a good experimental model for investigating the molecular physiology of ion transport in ameloblasts (Bori et al., 2016; Racz et al., 2017; Racz et al., 2018; Varga et al., 2018). Furthermore, this cell line has been successfully used in a large number of studies to investigate ameloblast function (Matsumoto et al., 2011; Zheng et al., 2013a; Jalali et al., 2017; Yang et al., 2018b; Takahashi et al., 2019).

In this report we demonstrate that HAT-7 cells can form spheroids similar to those previously obtained in primary cultures of ameloblast-lineage cells. We have compared their capacity to regulate intracellular pH and to show intracellular calcium responses to extracellular stimulation, to our previous findings in 2D culture. To explore the effects of fluoride exposure we have determined the dose at which fluoride exposure disturbs HAT-7 spheroid formation.

## Materials and Methods

### Cell Culture

HAT-7 cells were cultured in “control medium” consisting of DMEM/F12 Ham medium (Sigma-Aldrich, St. Louis, MO, United States) supplemented with 10% HyClone fetal bovine serum (Thermo Scientific, Waltham, MA, United States), 100 U/ml penicillin and 10 μg/ml streptomycin (Sigma-Aldrich). They were incubated in a humidified atmosphere containing 5% CO_2_ at 37°C. The cell medium was changed every other day and the cells were subcultured when the cell confluence reached 70–80%.

### 2D Culture on Permeable Supports

HAT-7 cells were seeded on permeable polyester Transwell culture inserts with 0.4 μm pore size and 1.12 cm^2^ surface area (Costar, Corning, NY, United States) and were cultured in “differentiation medium” consisting of control medium, as described above, supplemented with CaCl_2_ (2.1 mM final concentration) and 10^−8^ M dexamethasone (Sigma-Aldrich) (Arakaki et al., 2012; Bori et al., 2016).

### 3D Culture with Basement Membrane Matrix

For 3D culture, HAT-7 cells were grown within the gel layer of Matrigel^®^ Basement Membrane Matrix (Corning, NY, United States). For seeding at low density to obtain single cell derived colonies within the Matrigel/, approximately 80,000 HAT-7 cells were resuspended in 160 μl of each following media: 1) control medium, 2) differentiation medium, and 3) Hepato-STIM^®^ (BD Biosciences, San Jose, CA, United States), a commercially available epithelial selection medium, supplemented with 10% HyClone fetal bovine serum (Thermo Scientific), 1% l-glutamine (Sigma-Aldrich), 100 U/ml penicillin and 10 g/ml streptomycin (Bori et al., 2016). Each cell suspension was mixed with 400 μl of Matrigel and 70 μl aliquots were placed in eight individual wells of a 24-well Nunclon Sphera low-attachment plate (Thermo Fisher Scientific, Rochester, NY, United States). The plate was incubated at 37°C to allow the Matrigel to solidify, and then 1 ml of the appropriate medium was added to each well. The culture medium was changed every other day.

### Morphology

Spheroid development was assessed in living HAT-7 cell cultures by phase-contrast light microscopy (Nikon Eclipse TE200) and images obtained using a CCD camera (Blackfly USB3 CCD camera, FLIR Systems, Wilsonville, OR, United States). Size distribution was determined using the particle analysis function in ImageJ (NIH, Bethesda, MD, United States).

For histology, spheroids grown for 7 days in Matrigel with Hepato-STIM medium were released from the matrix using 0.25% trypsin/EDTA and fixed in 4% paraformaldehyde. Frozen sections were cut at 5 µm thickness and stained with haematoxylin and eosin.

### RT-PCR

Total RNA was isolated from 7-days HAT-7 spheroids and HAT-7 monolayers grown on Transwell permeable supports, using a NucleoSpin RNA XS kit (Macherey-Nagel). The integrity of the RNA was assessed by running the purified RNAs on 1% agarose gels. Only samples in which the amount of 28S rRNA was about twice the intensity of the 18 S rRNA were processed further. Total RNA (1.5 µg/sample) was reverse transcribed using a Maxima First Strand cDNA Synthesis Kit for RT-qPCR (Thermo Scientific). Amplification was performed using QuantStudio 5 with TaqPath qPCR Master Mix, CG (Applied Biosystems, Foster City, CA, United States) and predesigned primers (Life Technologies Magyarország Kft., Budapest): Klk4 (Rn01498536_m1), Cldn1 (Rn00581740_m1), Cldn4 (Rn01196224_s1), Cldn8 (Rn01767199_s1), Tjp1/ZO-1 (Rn02116071_s1), Slc4a2/AE2 (Rn00566910_m1), Slc4a4/NBCe1 (Rn00584747_m1), Slc9a1/NHE1 (Rn00561924_m1), CFTR (Rn01455971_m1), Slc26a4/pendrin (Rn00693043_m1). Acidic ribosomal protein P0 (RPLP0; Rn00821065_g1) was used as internal control. Each sample was measured in three technical parallels. No-template and RT-minus reactions were performed to monitor non-specific and genomic DNA amplifications respectively. Relative fold changes were calculated by the comparative Ct method (2−ΔΔCT).

### Microfluorometry

For measurement of intracellular pH (pH_i_) and calcium concentration ([Ca^2+^]_i_), spheroids cultured in Hepato-STIM medium for one week were released from the Matrigel, using 0.25% Trypsin/EDTA, and plated at low density on 25 mm diameter coverslips coated with 0.01% poly-l-lysine solution (Sigma-Aldrich).

For pH_i_ measurements, the cells were loaded with BCECF-AM (3 μM, Molecular Probes, Eugene, OR, United States) for 60 min at 37°C. Coverslips were then mounted in a purpose-built perfusion chamber and the spheroids were superfused at 0.9 ml/min with either a HEPES-buffered solution containing (in mM): 137 NaCl, 5 KCl, 1 CaCl_2_, 1 MgCl_2_, 10 D-glucose, and 10 HEPES, pH 7.4, gassed with 100% O_2_, or a bicarbonate-buffered bath solution containing (in mM): 116 NaCl, 25 NaHCO_3_, 5 KCl, 1 CaCl_2_, 1 MgCl_2_, 10 D-glucose, and 5 HEPES, pH 7.4, gassed with 5% CO_2_ in O_2_. BCECF fluorescence was measured photometrically at 5-s intervals on an inverted fluorescence microscope (Nikon Eclipse TE200) as described previously (Racz et al., 2017). The ratio of the fluorescence signals obtained with excitation at 490 and 440 nm was converted to pH_i_ using calibration data obtained with the nigericin/high potassium method (Thomas et al., 1979).

For [Ca^2+^]_i_ measurements, the cells were loaded with fura-2AM (4 μM, Molecular Probes) for 45 min at room temperature. The spheroids were then superfused in a custom-made, open perfusion chamber at 2 ml/min with a bath solution containing (in mM): 137 NaCl, 5 KCl, 2 CaCl_2_, 1 MgCl_2_, 10 HEPES, and 10 glucose, adjusted to pH 7.4. Fura-2 fluorescence was measured alternately at 340, and 380 nm on an upright fluorescence microscope (Nikon Eclipse E600) using a metal-halide lamp and an internal filter wheel (Lumen 220 Pro, Prior Scientific, Cambridge, United Kingdom) at 1.5-s intervals. Imaging was performed with a cooled sCMOS camera (Prime BSI, Teledyne Photometrics, Tucson, AZ, United States) controlled by NIS AR software (Nikon). Changes in [Ca^2+^]_i_ were calculated as the ratio of the emitted fluorescence at the two respective excitation wavelengths (F_340_/F_380_) and normalized to the baseline ratio.

### Fluoride Exposure

HAT-7 cells were plated in Matrigel and fed with Hepato-STIM medium as described above. There were two experimental groups. In the first group, the culture medium was changed 24 h after seeding to Hepato-STIM containing 0 (control), 0.1, 0.3, 1.0, or 3.0 mM fluoride. In the second group, the culture medium was changed to Hepato-STIM, containing the same range of fluoride concentrations, on day 4 when spheroids were beginning to form. In both groups the medium was refreshed every other day.

Cell growth and spheroid development within the Matrigel layer were assessed by bright-field light microscopy (Nikon Eclipse TE200) and images obtained using a CCD camera (Blackfly USB3 CCD camera, FLIR Systems).

### Statistical Analysis

Where appropriate, data are presented as mean ± SEM. Statistical analyses were performed using either one-sample t tests or analysis of variance combined with Tukey’s multiple comparison test as appropriate.

## Results

### HAT-7 Cells in 2D and 3D Culture

**FIGURE 1:**
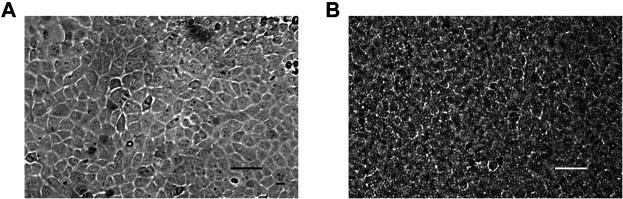
Morphology of 2D HAT-7 cell cultures. **(A)** HAT-7 cells grown in control medium as a conventional monolayer on plastic in a T-25 culture flask for 3 days. **(B)** HAT-7 cells grown in differentiation medium as a polarized monolayer on a Transwell permeable support for 7 days. Also visible are the 0.4 µm pores in the polyester supporting membrane. Scale bars: 100 µm.

For conventional 2D culture on plastic, HAT-7 cells were seeded in T-25 tissue culture flasks and incubated in control medium. As the cells became confluent, they established a fairly uniform polygonal shape, giving a cobblestone appearance typical of epithelial cells (Figure 1A). When grown on permeable Transwell supports, their appearance was similar, as shown in Figure 1B where the outlines of the 0.4 µm pores in the polyester filter can also be seen.

**FIGURE 2:**
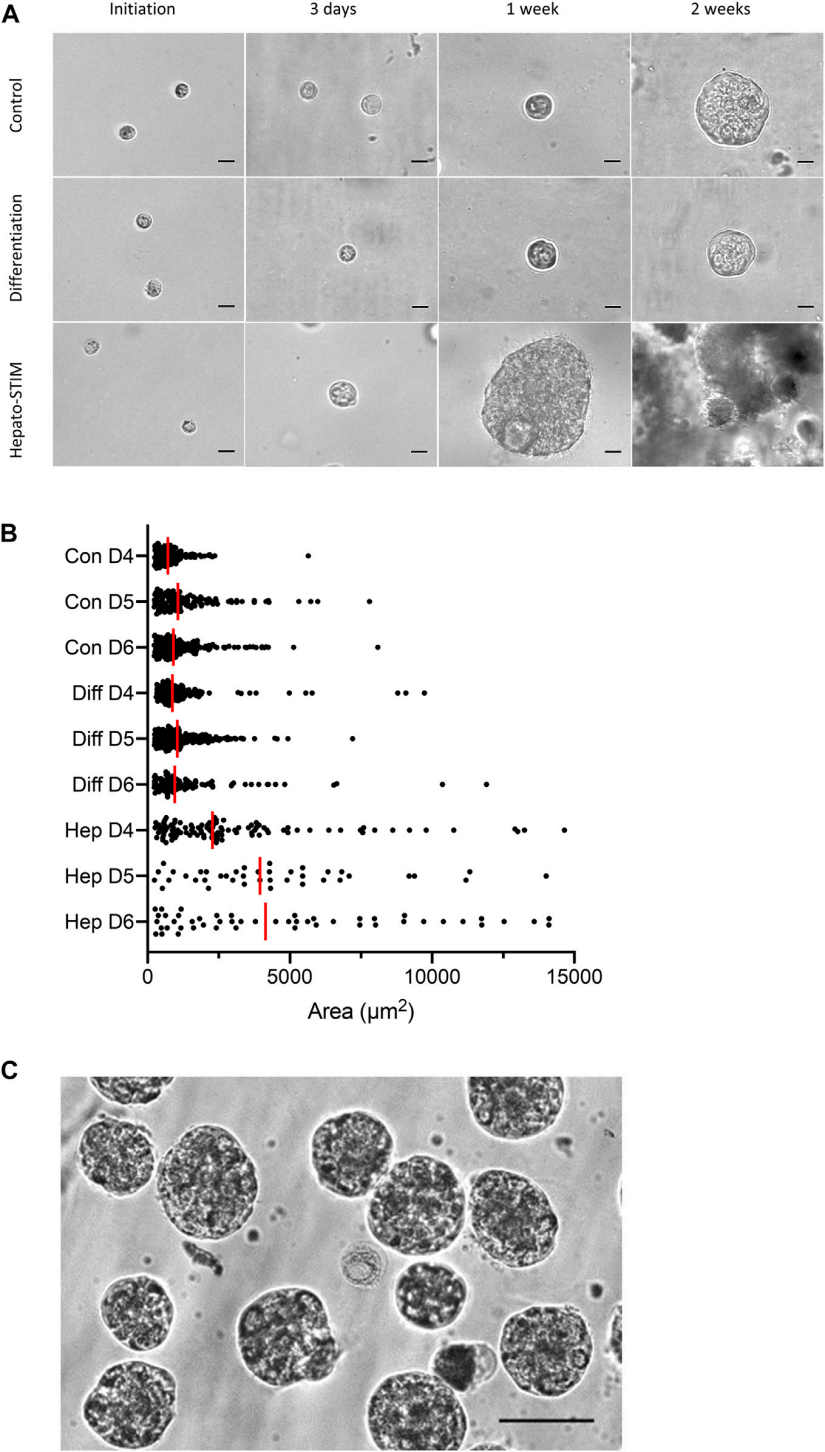
Morphology of 3D HAT-7 cell cultures grown within a Matrigel matrix. **(A)** Time courses of growth and spheroid development when HAT-7 cells were seeded in Matrigel matrix and incubated in three different culture media: control, differentiation and Hepato-STIM. Images were obtained on the day of seeding and then after 3 days, 1 week and 2 weeks. **(B)** Size distribution of HAT-7 cells/spheroids in a representative experiment where growth in the three different culture media was compared on days 4, 5, and 6 (D4-D6). Each point represents the area of a single cell/spheroid and the vertical red bars indicate the median values. **(C)** HAT-7 spheroids isolated from the Matrigel matrix after 7 days of culture in Hepato-STIM and then plated on glass coverslips coated with poly-l-lysine in preparation for microfluorometry experiments to measure intracellular pH and calcium. Scale bars: 20 µm **(A)**, 75 µm **(C)**.

For 3D culture within the extracellular matrix layer, fully dissociated HAT-7 cells were mixed with Matrigel prior to seeding in low-attachment plate wells. The cells, which were initially quite uniform in size, were then incubated with one of three alternative culture media and imaged after 3 days, 1 week and 2 weeks (Figure 2A). The cells cultured in control and differentiation media showed little change in appearance over the first 3 days. After 1 week some of the cells were began to show signs of division. And after 2 weeks a few had formed larger, multicellular spheroids, as depicted in the figure. However, these represented only a small fraction of the population, the rest remaining unchanged in size from their initial seeding.

In contrast, cells cultured in Hepato-STIM medium divided and formed multicellular spherical structures much more rapidly and consistently. Signs of cell division were already evident in most of the cell population after 3 days, and after just one week, many had developed into spheroids approaching 100 µm in diameter. After 2 weeks the spheroids had grown very large and had begun to disintegrate. The change in size distribution of the cells/spheroids over days 4, 5, and 6 (Figure 2B) is clearly much more rapid in Hepato-STIM compared with the other media. The yield and size of the spheroids after just one week in Hepato-STIM was much greater than in the control and differentiation media, even when the latter groups were cultured for 2 or more weeks. After culture in Hepato-STIM for one week, numerous HAT-7 spheroids could be released from the Matrigel matrix with trypsin/EDTA, resuspended in physiological salt solutions (Figure 2C) and used for a variety of experimental procedures.

**FIGURE 3:**
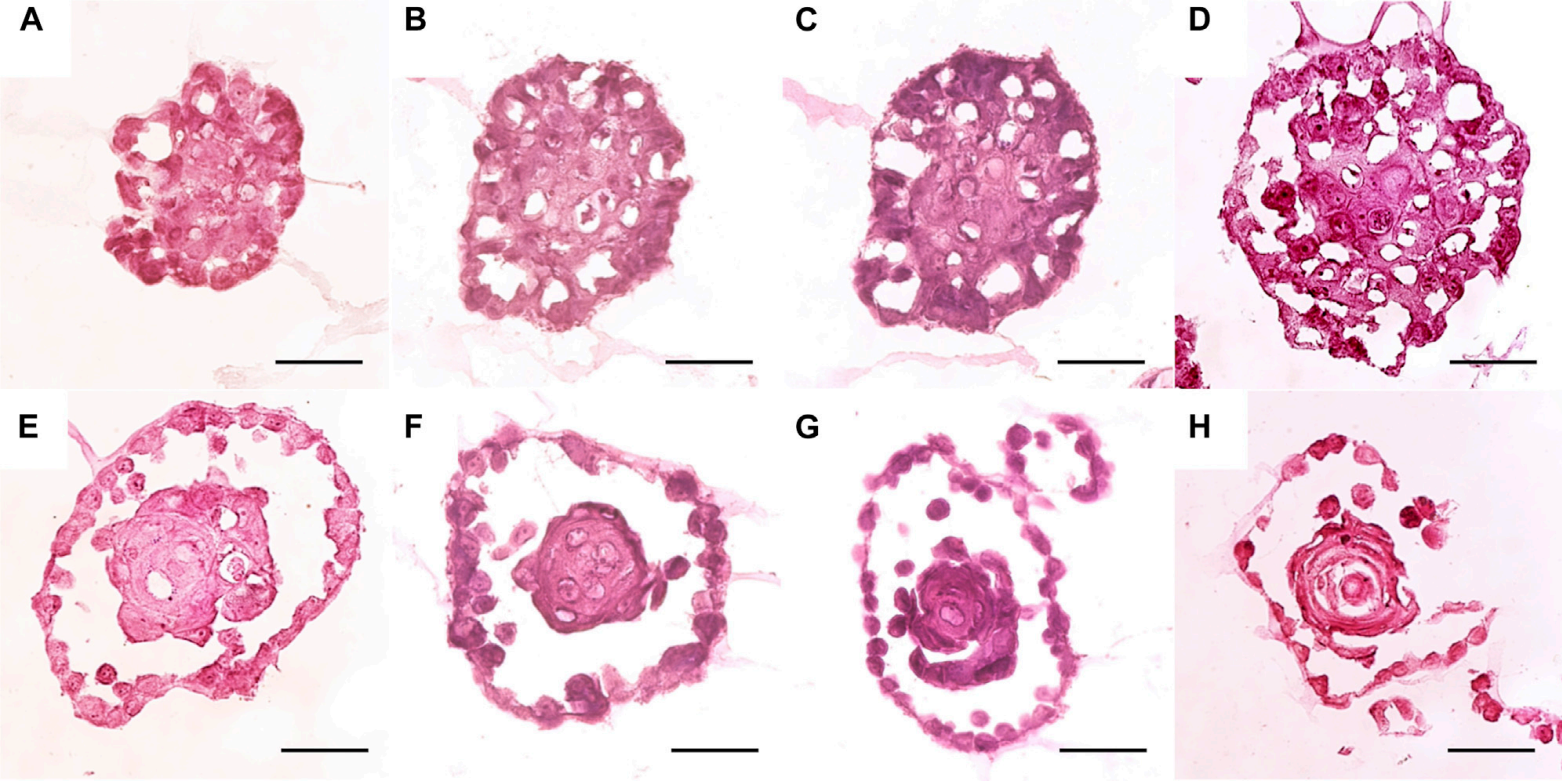
Histology of HAT-7 spheroids grown for 7 days in Matrigel with Hepato-STIM medium. Spheroids were isolated from the matrix using 0.25% trypsin/EDTA. Frozen sections (5 µm) were stained with haematoxylin and eosin. Scale bars: 50 µm.

The internal structure of the HAT-7 spheroids, cultured in Matrigel with Hepato-STIM medium, was examined in frozen sections stained with haematoxylin and eosin (Figure 3). After 7 days in culture, a range of morphologies was observed. The smallest and simplest spheroids consisted of a dense mass of largely undifferentiated cells with occasional extracellular fluid spaces or “lacunae” (Figure 3A). At the other end of the range were spheroids consisting of a central cell mass separated from an outer epithelial monolayer by a clear fluid-filled space, thereby showing various degrees of lumen formation (Figures 3E–H). The central cell mass could be either disorganized and undifferentiated (Figures 3E,F) or lamellar (Figures 3G,H) in structure. The spheroids with an intermediate morphology (Figures 3B–D) contained significant numbers of lacunae and evidence of an emerging outer epithelial layer that had not yet completely separated from the central cell mass.

### RT-qPCR

**FIGURE 4:**
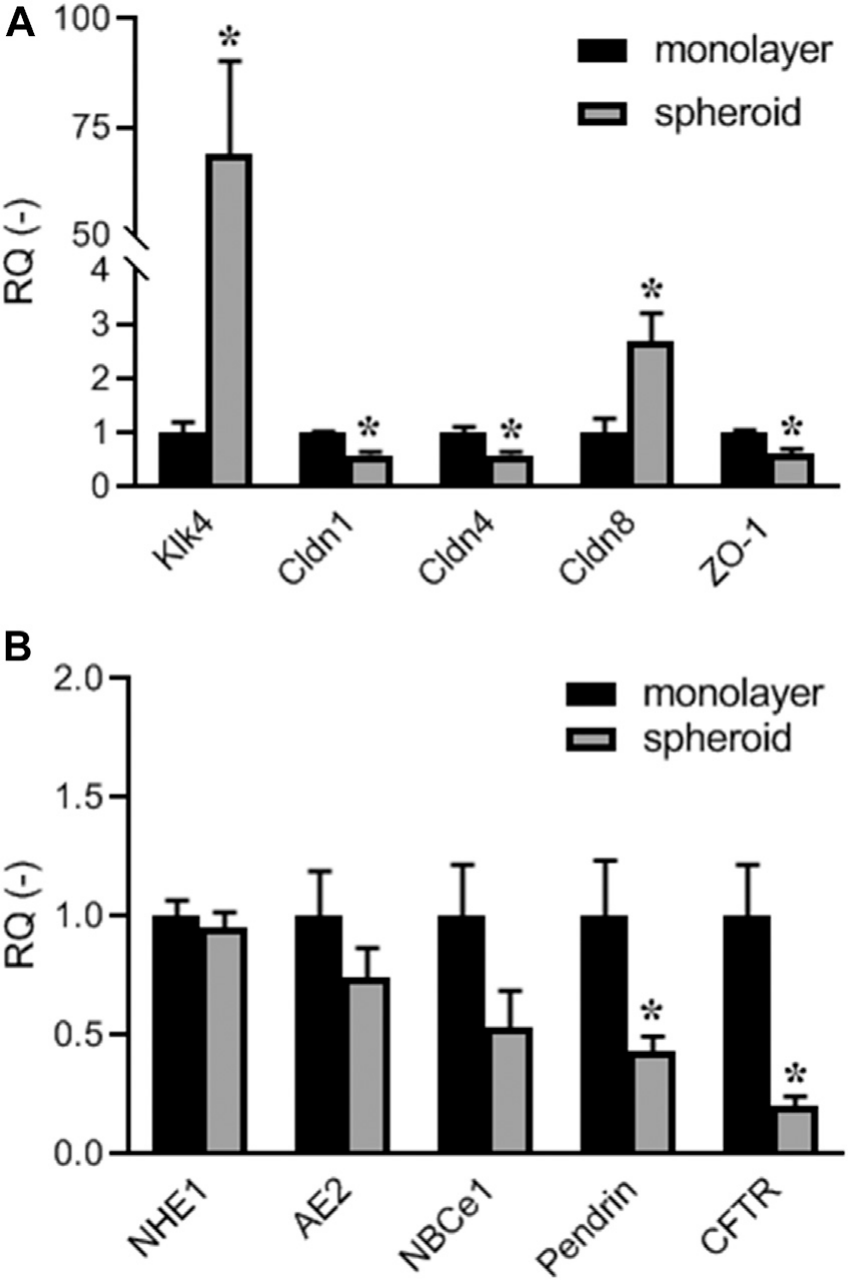
Quantitative RT-PCR data showing expression of: **(A)** maturation-phase ameloblast marker Klk4, and tight-junction protein genes Cldn1, Cldn4, Cldn8 (claudins 1, 4, and 8), and Tjp1 (ZO-1); **(B)** electrolyte transporter genes Slc9a1 (NHE1), Slc4a2 (AE2), Slc4a4 (NBCe1), Slc26a4 (pendrin), and Cftr (CFTR). Expression levels in HAT-7 cells grown in Matrigel with Hepato-STIM medium (spheroid) were normalised to expression levels in HAT-7 cells grown on Transwell permeable supports in differentiation medium (monolayer). Data are mean ± SEM. *p < 0.05 (n = 4).

To examine the expression levels of key structural, functional and marker genes, quantitative RT-PCR was performed using mRNA extracted from HAT-7 spheroids grown in Matrigel with Hepato-STIM medium, and from HAT-7 monolayers grown on Transwell permeable supports. Expression levels in the spheroids were normalised to the monolayer data (Figure 4) in order to reveal any significant differences between the 3D spheroids and the 2D monolayers used in our previous functional studies (Bori et al., 2016; Racz et al., 2017).

The most striking difference was in the expression of the maturation-stage ameloblast marker Klk4, which was increased approximately 70-fold in the HAT-7 spheroids compared with the monolayers (Figure 4A). There were significant decreases in the tight-junction proteins claudin-1, claudin-4 and TJP1/ZO-1, and an increase in claudin-8. Looking at the electrolyte transporters that are involved in HCO_3_^-^ secretion by HAT-7 cells (Figure 4B), there were significant reductions in the expression of SLC26A4/pendrin and CFTR (cystic fibrosis transmembrane conductance regulator) in the spheroids–both normally associated with the apical membrane of ameloblasts. Small decreases in the expression of the basolateral transporters NHE1, AE2, and NBCe1 were not statistically significant.

### Intracellular pH Regulation

Functional activities of the key transporters involved in HCO_3_^-^ secretion by HAT-7 cells were assessed using BCECF fluorescence to measure intracellular pH changes in small groups of HAT-7 spheroids grown in Matrigel and Hepato-STIM. Standard protocols were used to evaluate the contributions of individual transporters previously shown to be active in 2D cultures of HAT-7 cells (Bori et al., 2016; Racz et al., 2017).

**FIGURE 5:**
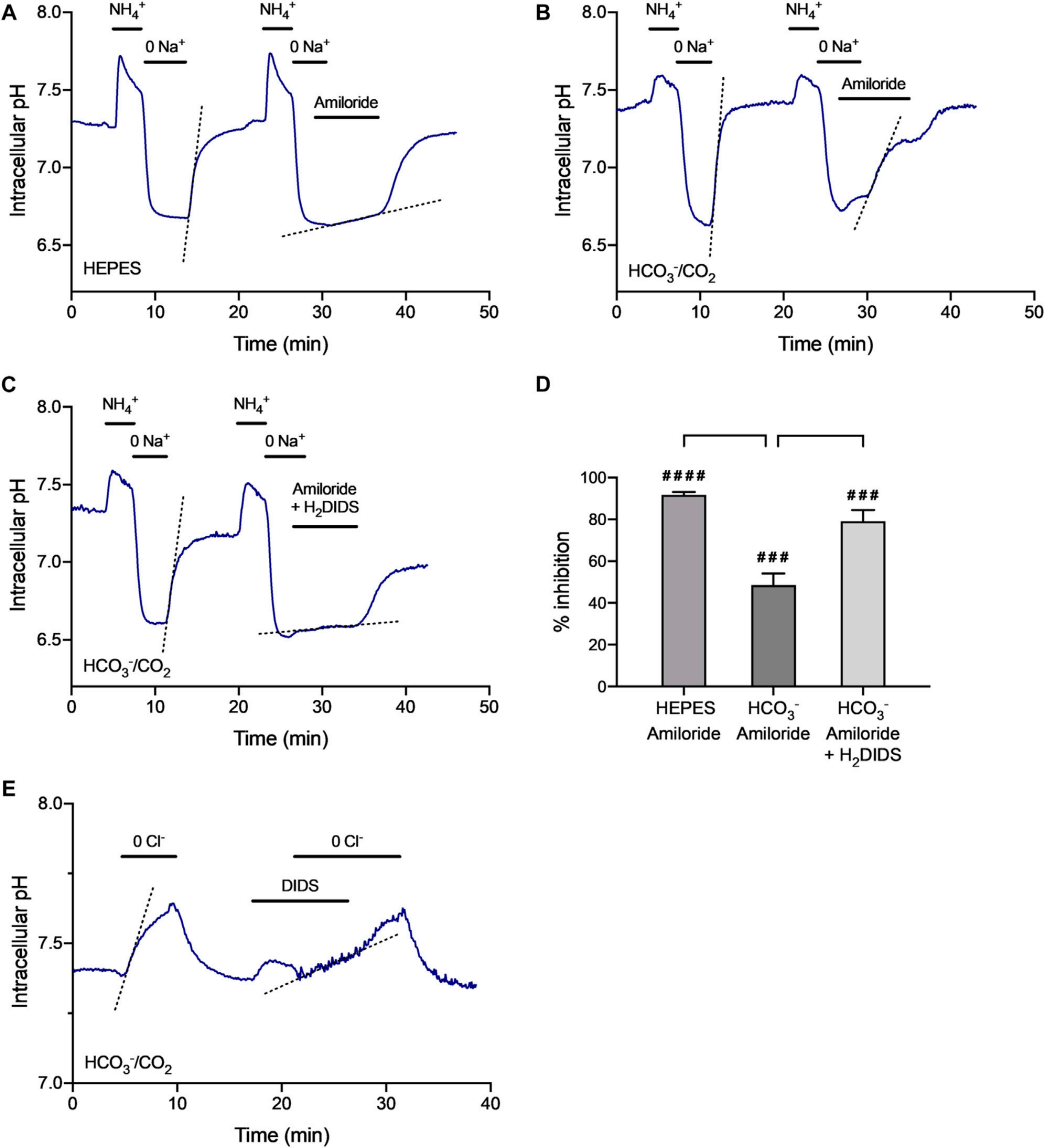
**|** Regulation of intracellular pH in isolated HAT-7 spheroids. **(A–C)** Recovery of pH_i_ from intracellular acidification evoked by a 3-min exposure to extracellular NH_4_^+^ (20 mM) followed by substitution of extracellular Na^+^ with NMDG^+^ (*0 Na* ^*+*^): **(A)** in HEPES-buffered bath solution in the absence and presence of the Na^+^/H^+^ exchange inhibitor amiloride (0.3 mM); **(B)** in HCO_3_^-^-buffered bath solution in the absence and presence of amiloride (0.3 mM); **(C)** in HCO_3_^-^-buffered bath solution in the absence and presence of amiloride (0.3 mM) together with the Na^+^-HCO_3_^−^ cotransport inhibitor H_2_DIDS (0.5 mM). Dashed lines show the rate of recovery of pH_i_ when Na^+^ was restored to the perfusate. **(D)** Averaged data (mean ± SEM) from 4–6 experiments showing the inhibitory effects of amiloride and H_2_DIDS on the rate of recovery of pH_i_ from acidification in the absence and presence of HCO_3_^−^. Percentage inhibition was calculated with reference to the internal control in each experiment. ####*p* < 0.0001, ###*p* < 0.001 compared with control (one-sample t test). **p < 0.01, ***p < 0.001 (ANOVA and Tukey post-hoc test). **(E)** Changes in pH_i_ evoked by substitution of extracellular Cl^−^ with gluconate^-^ (*0 Cl* ^−^) in HCO_3_^−^-buffered bath solution in the absence and presence of DIDS (0.1 mM). Dashed lines show the rate of recovery of pH_i_ when Na^+^ or Cl^−^ was restored to the perfusate. Measurements of BCECF fluorescence were pooled from 20–50 HAT-7 spheroids isolated from Matrigel matrix by treatment with 0.25% trypsin/EDTA. Traces are representative of 4–6 experiments.

Recovery of pH_i_ from intracellular acidification was examined by exposing HAT-7 spheroids to NH_4_Cl (20 mM) for 3 min, followed by substitution of extracellular Na^+^ with NMDG^+^. In a HCO_3_^-^-free, HEPES-buffered bath solution this resulted in a transient alkalinization of the cells (due to NH_3_ entry) followed by a rapid drop in pH_i_ to a markedly acidic value (Figure 5A). This acidification was sustained in the continuing absence of Na^+^ indicating that there was no significant Na^+^-independent pathway for H^+^ extrusion from the cells, such as an H^+^-ATPase. When Na^+^ was restored to the bath, pH_i_ recovered rapidly to control values, indicating the presence of an Na^+^-dependent H^+^ extruder. When Na^+^ was restored in the presence of amiloride (0.3 mM)–a selective inhibitor of Na^+^/H^+^ exchange–the rate of recovery from acidification was greatly reduced. This indicates that an Na^+^/H^+^ exchanger, most probably NHE1, is active in HAT-7 spheroids. Averaged data for the percentage inhibition of the rate of recovery, compared with the corresponding control in each experiment, is shown in Figure 5D.

When the experiment was repeated in the presence of HCO_3_^-^, the recovery from acidification, when Na^+^ was restored to the bath, was less effectively inhibited by amiloride alone (Figures 5B,D), indicating the likely contribution of an additional Na^+^- and HCO_3_^−^-dependent mechanism. The recovery of pH_i_ in these conditions was more effectively inhibited (Figures 5C,D) by amiloride applied in combination with H_2_DIDS (0.5 mM)-an inhibitor of Na^+^-HCO_3_^-^ cotransport. It is therefore likely that the HAT-7 spheroids also express an active Na^+^-HCO_3_^−^ cotransporter, most probably NBCe1.

The activity of Cl^-^/HCO_3_^-^ exchangers in HAT-7 spheroids was examined by substitution of extracellular Cl^-^ with gluconate^-^. In the HCO_3_^-^-buffered bath solution, Cl^−^ substitution evoked a significant rise in pH_i_ in the spheroid cells (Figure 5E), most probably as a result of Cl^−^ efflux driving an influx of HCO_3_^−^ by Cl^-^/HCO_3_^-^ exchange. This rise in pH_i_ was completely reversed when Cl^−^ was restored to the bath solution. Furthermore, pre-treatment with DIDS (0.1 mM) - an inhibitor of Cl^−^/HCO_3_^−^ exchange-reversibly reduced the rate of change in pH_i_ resulting from Cl^-^ substitution by 72.1 ± 12.1% (n = 4, *p* < 0.01). This indicates that an exchanger of this type, most probably AE2, is active in HAT-7 spheroids.

### Calcium Signaling

Previous work has shown that transepithelial HCO_3_^−^ secretion by HAT-7 cells in 2D culture is stimulated by agonists known to elevate intracellular cAMP and Ca^2+^, such as forskolin and ATP respectively (Bori et al., 2016). In the present study, changes in intracellular Ca^2+^ concentration ([Ca^2+^]_i_) were measured in HAT-7 spheroids using fura-2 fluorescence.

**FIGURE 6:**
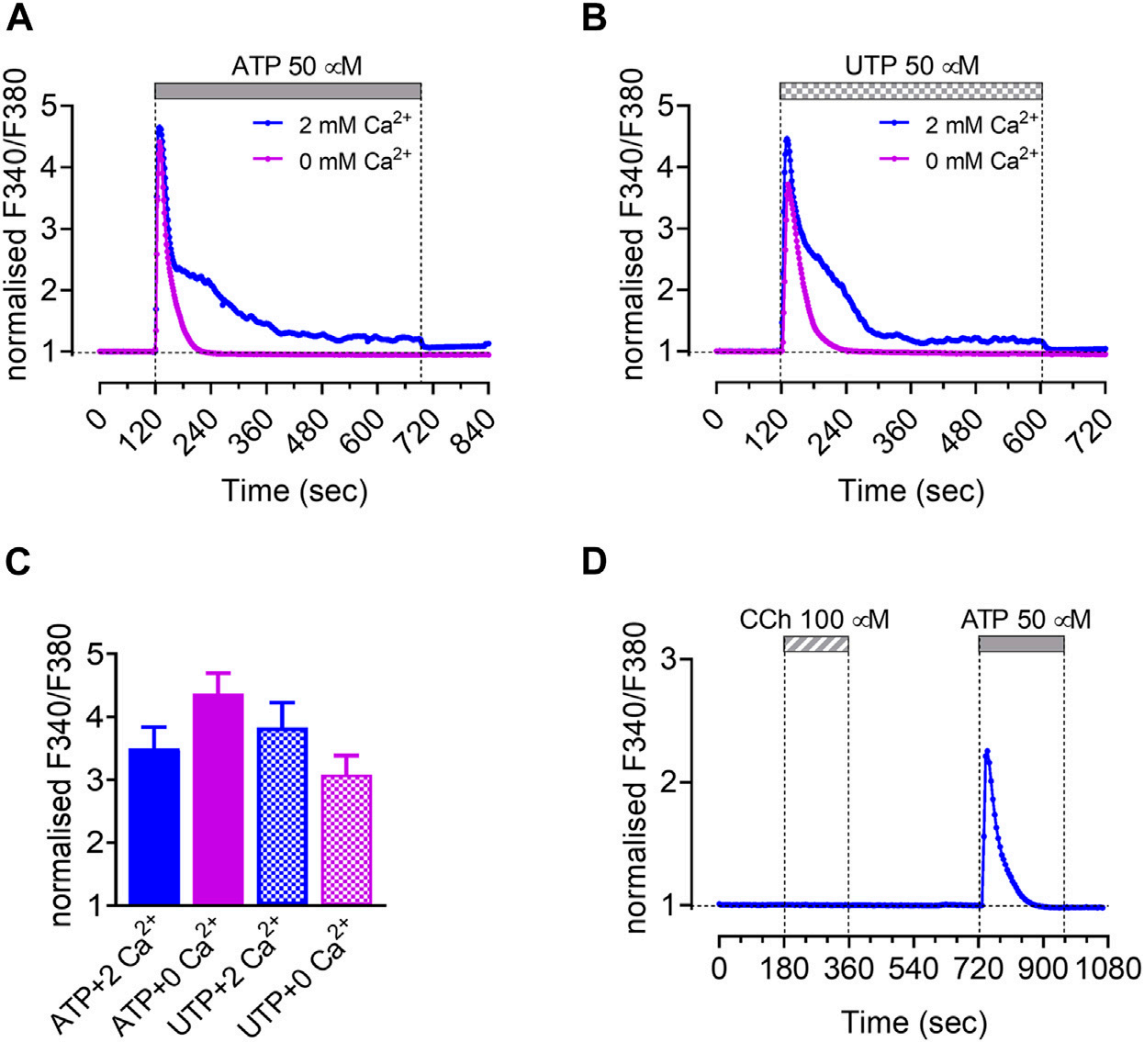
Intracellular Ca^2+^ responses of isolated HAT-7 spheroids to different agonists. Changes in intracellular Ca^2+^ in response to 50 μM ATP (n = 5) **(A)** and 50 μM UTP (n = 3) **(B)** in the presence and absence of extracellular calcium. **(C)** Peak values presented as means ± SEM (no significant difference). **(D)** Effect of carbachol (100 μM, n = 3) on intracellular Ca^2+^ concentration. An ATP stimulus was applied subsequently to test the responsiveness of the spheroids. Panels show representative traces. Data are presented as changes in fura-2 fluorescence ratio normalized to baseline.

To test for the presence of purinergic receptors in the spheroids, we measured the intracellular Ca^2+^ concentration [Ca^2+^]_i_ after stimulation with the purinergic agonists ATP (50 µM) and UTP (50 µM). In the presence of external Ca^2+^ (2 mM) ATP and UTP induced similar biphasic Ca^2+^ responses consisting of an initial transient peak and a sustained component. However, in the absence of external Ca^2+^, both ATP and UTP caused only transient Ca^2+^ responses, suggesting that the sustained component was due to Ca^2+^ influx from the extracellular space (Figures 6A–C). Since UTP does not stimulate ionotropic P2X receptors, we speculate that Ca^2+^ entry was due to depletion of intracellular Ca^2+^ stores, which in turn activates store-operated Ca^2+^ channels (SOCCs).

Since acetylcholine (ACh) has been shown to elicit Ca^2+^ signals in freshly isolated rat ameloblasts (Nurbaeva et al., 2018), we also tested the effects of the Ach analog carbachol (100 µM). However, no changes were observed in [Ca^2+^]_i_ suggesting that neither muscarinic nor nicotinic functional ACh receptors are expressed in HAT-7 spheroids (Figure 6D).

### Fluoride Exposure

The effects of fluoride on spheroid formation in the Matrigel matrix were examined in two series of experiments where fluoride, at a range of concentrations, was introduced at two different time points during the culture process.

**FIGURE 7:**
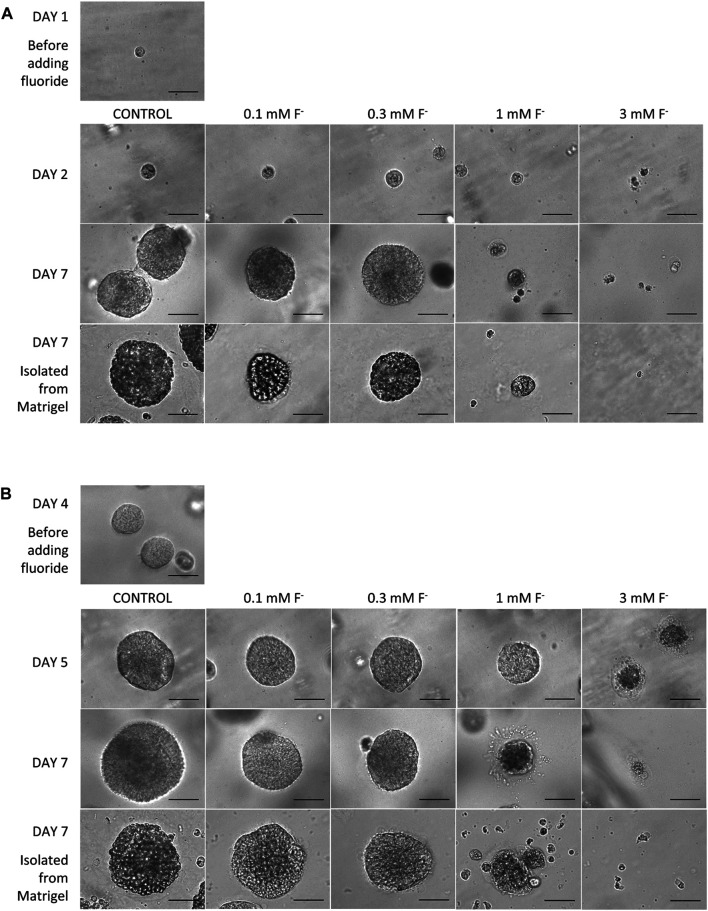
Morphology of HAT-7 spheroids cultured in Matrigel and Hepato-STIM medium in the absence (control) or presence of fluoride (0.1–3.0 mM). **(A)** Spheroid images obtained on days 1, 2, and 7 when fluoride (0, 0.1, 0.3, 1.0, 3.0 mM) was added to the culture medium 24 h after seeding. Also shown are spheroids isolated from the Matrigel on day 7. **(B)** Spheroid images obtained on days 4, 5, and 7 when fluoride (0, 0.1, 0.3, 1.0, 3.0 mM) was added to the culture medium at the time of rapid spheroid growth 4 days after seeding. Also shown are spheroids isolated from the Matrigel on day 7. Scale bars: 70 µm.

In the first series, fluoride was added to the Hepato-STIM medium 24 h after the cells had been seeded in Matrigel. The concentrations of fluoride applied were 0.1, 0.3, 1.0, and 3.0 mM, and there was a control group that was not exposed to fluoride. Figure 7A shows images of the developing spheroids obtained on days 2 and 7 of culture, and images of spheroids isolated from the Matrigel matrix on day 7. In the absence of fluoride (control), the spheroids developed quickly and uniformly as already seen in Figure 2A. HAT-7 cells cultured in 0.1 and 0.3 mM fluoride were also able to form spheroids, with a similar size and appearance to the control group, over a similar time period. When these spheroids were isolated from the Matrigel on day 7, they retained the same shape and appearance as the control group. In other words, spheroid formation did not appear to be significantly affected by 0.1 or 0.3 mM fluoride.

In contrast, HAT-7 cells cultured in Matrigel and Hepato-STIM in the presence of 1 mM fluoride generated only a few small spheroids and significant amounts of cellular debris. The spheroids isolated from the Matrigel on day 7 were few in number and smaller than those obtained at the lower fluoride concentrations and in the control group. As expected, in 3 mM fluoride there were few viable cells remaining after a single day, and no spheroids were formed.

In the second series, fluoride was introduced on day 4, which is the time point when the spheroids start to grow rapidly in Matrigel and Hepato-STIM under control conditions. As can be seen in Figure 7B, HAT-7 spheroid development was again largely unaffected by 0.1 and 0.3 mM fluoride. The number, growth rate and final size of the spheroids was similar to those grown in control medium. The higher concentrations of fluoride applied on day 4 had similar effects to what was observed when fluoride was applied after 24 h. The spheroids already present on day 4 gradually decreased in size in the presence of 1 mM fluoride, and by day 7 they were clearly disaggregating. This was particularly noticeable when they were isolated from the Matrigel matrix: few intact spheroids remained and there was a lot of cell debris. In 3 mM fluoride, the death and disintegration of the spheroids proceeded more rapidly and there were none remaining by day 7.

## Discussion

In the present study dissociated HAT-7 cells were cultivated within a Matrigel matrix in order to obtain single cell-derived 3D colonies. We found that the combination of Hepato-STIM epithelial cell differentiation medium and Matrigel induced cell expansion and 3D spheroid formation. The cells retained epithelial cell morphology and continued to express both ameloblast-specific and ion transport-specific gene expression. Furthermore, like HAT-7 polarized monolayers, the spheroid cells were able to regulate their intracellular pH and to show intracellular calcium responses to extracellular stimulation. Finally, we have demonstrated that HAT-7 spheroids may serve as a disease model for studying the concentration-dependent damaging effects of fluoride exposure on amelogenesis.

The choice of culturing protocols, culture medium and scaffold serve as important selection factors in epithelial cell expansion and 3D culturing (Bajaj et al., 2014). We initially tested three culture media: control medium (standard DMEM/F12 Ham medium supplemented with fetal bovine serum), differentiation medium (control medium supplemented with CaCl_2_ and dexamethasone) and Hepato-STIM medium (also supplemented with fetal bovine serum). It quickly became apparent that the use of Hepato-STIM medium, rather than the other two media, resulted in a far better outcome, both regarding the number and size of the HAT-7 spheroids. Hepato-STIM is a specialised medium consisting of Williams’ E medium supplemented with dexamethasone, insulin, transferrin, selenium, EGF and 1.8 mM Ca^2+^. These components are all important factors in maintaining an epithelial phenotype (Szlavik et al., 2008a; Maria and Tran, 2011; Hegyesi et al., 2015). This medium was originally designed for the culture of hepatocytes, but has since been used successfully to grow both human primary salivary epithelial cells (Szlavik et al., 2008a; Maria and Tran, 2011; Hegyesi et al., 2015) and lacrimal acinar cells (Schonthal et al., 2000; Tiwari et al., 2018).

In the present work we used Matrigel as a scaffold to induce and promote spheroid formation by HAT-7 cells. Matrigel is a poorly defined basement membrane derivative extracted from Engelbreth–Holm–Swarm mouse sarcoma cells, thus its direct application in regenerative medicine is limited (Huang et al., 2020). However, its use is well established in the culture of spheroids and organoids from various tissues, especially those of epithelial origin. This is mainly because of three key factors: first, the Matrigel provides anchoring molecules such as the arg-gly-asp (RGD) sequence for cell adhesion; second, it has optimal stiffness for housing the cells; and third, it contains laminin-111, an important extracellular component that can independently provide biological signals for spheroid/organoid formation and growth (Huang et al., 2020). Using Matrigel, 3D spheroids and organoids have been created from intestinal epithelial cells (Huang et al., 2019), pancreas (Bakhti et al., 2019; Molnar et al., 2020), lacrimal glands (Schonthal et al., 2000; Massie et al., 2018) and salivary glands (Szlavik et al., 2008a; Szlavik et al., 2008b; Maria et al., 2011). Primary culture ameloblast-lineage cells also form 3D spheroids when cultured in Matrigel (Li et al., 2006).

When porcine and human ameloblast-lineage cells (Li et al., 2006; He et al., 2010) are grown in Matrigel, the cells form spherical, acinar-like structures that appeared similar to enamel pearls. Additionally, ameloblast-lineage cells (He et al., 2010) or human embryonic stem cell-derived epithelial cells (Zheng et al., 2013b; Zheng et al., 2013c) pre-cultivated in Matrigel and then cultured together with dental mesenchymal cells result in the formation of tooth-like structures. Finally, when mouse dental epithelial stem cells from the most posterior part of the cervical loop of incisors are isolated, dispersed, and then embedded into Matrigel (Natsiou et al., 2017) spheroids are produced (Natsiou et al., 2017). Our present success in reliably and reproducibly generating 3D HAT-7 spheroids, using Hepato-STIM culture medium and a Matrigel scaffold, are completely in line with those previous observations.

Tight junction proteins ZO-1 and claudins 1, 4, and 8 were all expressed at mRNA level in HAT-7 cells, regardless of whether they were grown on porous Transwell filters or in Matrigel. PCR data normalized to 2D HAT-7 cultivation revealed an increase in Cldn8 while the expression of ZO-1, Cldn1 and Cldn4 claudins were decreased in HAT-7 spheroids. These data are also in accordance with previous studies showing the expression of Cldn1, Cldn4 and Cldn8 in maturation-stage ameloblasts (Inai et al., 2008; Hata et al., 2010). The higher level of Cldn8 expression in the spheroids may reflect the elevated level of paracellular leakage observed when Cldn8 is upregulated (Amasheh et al., 2009).It is important to note that the expression of maturation ameloblast-specific Klk4 was considerably increased, suggesting a shift toward the maturation-type phenotype during 3D organization (Bronckers et al., 2016; Nunez et al., 2016).

The key electrolyte transporters and channels NHE1, AE2, NBCe1, pendrin and CFTR, which are key factors in HCO_3_^−^ secretion and intracellular pH regulation, were all expressed in both HAT-7 monolayers and spheroids, although pendrin and CFTR were significantly decreased in the spheroids. Nonetheless these data are generally in line with previous studies which demonstrated NHE1, AE2, NBCe1, pendrin and CFTR expression in ameloblasts and their involvement in intracellular and extracellular pH regulation (Bronckers et al., 2011; Lacruz et al., 2013; Jalali et al., 2014). The fact that these transporters are present in HAT-7 spheroids suggests that this experimental model, like the polarized 2D HAT-7 monolayer, is suitable for studying ameloblast acid/base transport.

An important function of ameloblasts is to produce hydroxyapatite crystals, comprising Ca^2+^, phosphate and water, in a process whereby eight moles of protons are liberated during the formation of each mole of hydroxyapatite (Lacruz et al., 2017; Varga et al., 2018). While the Ca^2+^ transport mechanism in these cells is relatively well understood (Lacruz, 2017; Lacruz et al., 2017; Varga et al., 2018), much less is known about the mechanism of phosphate transport into the enamel matrix. Some possibilities include transport into the cells of the enamel via Na^+^-phosphate cotransporters of the SLC20 and SLC34 gene families (Merametdjian et al., 2018; Beck, 2019) followed by secretion across the apical membrane or into matrix vesicles (Pandya et al., 2017).

Functional models are needed to better understand how ameloblasts direct Ca^2+^ and phosphate transport into the enamel matrix, and how ameloblasts neutralize the acidity generated as hydroxyapatite crystals form (Lacruz et al., 2017; Varga et al., 2018). HAT-7 cells grown as monolayers on permeable supports express ion transport proteins that mediate the uptake of HCO_3_^-^ ions across the basolateral membrane in order to drive HCO_3_^-^ secretion across the apical membrane (Bori et al., 2016; Racz et al., 2017). Basolateral HCO_3_^-^ uptake is achieved by the H_2_DIDS-sensitive Na^+^-HCO_3_^-^ cotransporter NBCe1, and indirectly by H^+^ extrusion via the amiloride-sensitive Na^+^/H^+^ exchanger NHE1 works in conjunction with intracellular carbonic anhydrase activity continuously producing H^+^ and HCO_3_^-^. The expansion of these cells into organoid culture systems is an important step toward further studies of ameloblast-mediated ion transport and matrix mineralization.

The pH_i_ measurements presented here confirm that these transporters are also active in HAT-7 spheroids and that, as in the HAT-7 monolayers, there is no evidence of a basolateral H^+^-ATPase contributing to the uptake of HCO_3_^-^ ions. The Cl^-^ substitution experiments confirm the expression of a DIDS-sensitive Cl^-^/HCO_3_^-^ exchanger, most probably AE2, that can contribute to the basolateral uptake of Cl^-^ ions, which are also required for enamel formation (Bronckers et al., 2015b; Bronckers, 2017). And the RT-qPCR data confirm that all three transporters, NBCe1, NHE1, and AE2, are expressed in HAT-7 spheroids as they are in HAT-7 monolayers.

It was interesting to note that CFTR expression decreased in spheroid culture. *In vivo*, CFTR is highly expressed in maturation-stage ameloblasts with reduced expression in the earlier transition stage and almost no expression in the secretory stage (Bronckers et al., 2010; Bronckers et al., 2015a). In previous studies of primary ameloblast-derived cells in grown in Matrigel, we found increased expression and synthesis of amelogenin protein (He et al., 2010). Amelogenin is a secretory stage protein, and this suggests the possibility that in 3D culture, ameloblast-lineage cells may de-differentiate to an early stage of enamel formation. Further studies and culture media can be used to explore this interesting possibility.

Calcium signaling in HAT-7 spheroids was also investigated in the present study. According to the current classification, adenosine stimulates P1 purinergic receptors whereas the extracellular nucleotides ATP, ADP, UTP, and UDP activate P2 receptors, which are further divided into two subtypes: ionotropic (P2X) and metabotropic (P2Y). In the presence of external Ca^2+^, both ATP and UTP elicited biphasic Ca^2+^ responses in HAT-7 spheroids, suggesting the activation of metabotropic P2Y2 and/or P2Y4 receptors (Guns et al., 2005). Furthermore, it has been demonstrated that P2Y receptors could regulate the activity of the NCKX4 Ca^2+^ extrusion pathway (Yang and Lytton, 2013) that is known to play a critical role in dental enamel maturation (Nurbaeva et al., 2017). Since ionotropic P2X receptors are insensitive to UTP, the Ca^2+^ entry during the sustained phase is most likely due to opening of store-operated Ca^2+^ channels. Indeed Nurbaeva and colleagues have recently shown that ATP strongly stimulates store-operated Ca^2+^ entry in maturation but not secretory ameloblasts (Nurbaeva et al., 2018).

Environmental factors, such as exposure to excessive amounts of fluoride, can alter enamel formation. Most studies using ameloblast cell lines have found effects of fluoride only at millimolar concentrations (Li et al., 2006), whereas in primary ameloblast cell cultures fluoride can alter cell characteristics at micromolar levels (Yan et al., 2007; Zhang et al., 2007). In this study, we found that in the presence of 1 mM fluoride applied from day 4, spheroid size gradually decreased, and by day 7 they were clearly disaggregating. Longer term exposure to 1 mM fluoride, between days 2 and 7, resulted in the formation of only a few small spheroids and significant amounts of cellular debris. 3 mM fluoride killed all HAT-7 cells within 2 days.

Exposure to 0.1 and 0.3 mM fluoride for three or six days did not affect spheroid formation compared to controls. These findings are in line with our recent studies on the effect of fluoride using the 2D HAT-7 model, where cells were grown in monolayers on Transwell filters (Racz et al., 2017).

The delay in tight junction formation observed in the 2D monolayer model (Racz et al., 2017) also fits well with our finding that HAT-7 spheroids disintegrated after 3 days of exposure to 1 mM fluoride when isolated from Matrigel using even the most gentle spheroid isolation procedure. The data indicate that 1 mM fluoride not only affects spheroid formation, but it also weakens the cell-cell adhesion structures responsible of maintaining the spheroid structure.

Studies of the effects of micromolar, physiologic levels of fluoride on primary ameloblast culture show that, while there are effects on cell proliferation and apoptosis, there are few to no changes in gene expression (Kubota et al., 2005; Zhang et al., 2006; Yan et al., 2007; Zhang et al., 2007; Sharma et al., 2008; Zhang et al., 2016).

These findings highlight the importance of developing a robust and reproducible culture system to investigate questions such as how fluoride alone, or in combination with other environmental stressors, alters enamel formation and biomineralization. Our finding that HAT-7 cells can form spheroids similar to those formed by primary culture ameloblasts offers the possibility of scaling up this model system to better understand cell-cell and cell-matrix interactions and their importance in enamel formation.

In conclusion, this model system shows that HAT-7 cells cultivated within a Matrigel extracellular matrix form three-dimensional, multi-cellular, spheroidal structures that retain their functional capacity for pH regulation and intracellular Ca^2+^ signaling. They also express protein markers consistent with a maturation-stage ameloblast phenotype. This new 3D model will allow us to gain a better understanding of the molecular mechanisms involved in amelogenesis, not only in health but also in disorders of enamel formation, such as those resulting from environmental factors.

## Data Availability

The raw data supporting the conclusions of this article will be made available by the authors, without undue reservation.
